# Evolution and Genetic Diversity of Primate Cytomegaloviruses

**DOI:** 10.3390/microorganisms8050624

**Published:** 2020-04-25

**Authors:** Rachele Cagliani, Diego Forni, Alessandra Mozzi, Manuela Sironi

**Affiliations:** Scientific Institute, IRCCS E. MEDEA, Bioinformatics, 23842 Bosisio Parini, Italy

**Keywords:** cytomegalovirus, non–human primates, genome organization, species–specificity, positive selection

## Abstract

Cytomegaloviruses (CMVs) infect many mammals, including humans and non–human primates (NHPs). Human cytomegalovirus (HCMV) is an important opportunistic pathogen among immunocompromised patients and represents the most common infectious cause of birth defects. HCMV possesses a large genome and very high genetic diversity. NHP–infecting CMVs share with HCMV a similar genomic organization and coding content, as well as the course of viral infection. Recent technological advances have allowed the sequencing of several HCMV strains from clinical samples and provided insight into the diversity of NHP–infecting CMVs. The emerging picture indicates that, with the exclusion of *core* genes (genes that have orthologs in all herpesviruses), CMV genomes are relatively plastic and diverse in terms of gene content, both at the inter– and at the intra–species level. Such variability most likely underlies the strict species–specificity of these viruses, as well as their ability to persist lifelong and with relatively little damage to their hosts. However, *core* genes, despite their strong conservation, also represented a target of adaptive evolution and subtle changes in their coding sequence contributed to CMV adaptation to different hosts. Indubitably, important knowledge gaps remain, the most relevant of which concerns the role of viral genetics in HCMV–associated human disease.

## 1. Introduction

*The family Herpesviridae* includes a variety of enveloped double–stranded DNA viruses that infect many vertebrates, including small mammals, humans, and non–human primates (NHPs) [1,2]. The family is divided into three subfamilies (*Alpha–*, *Beta–*, and *Gammaherpesvirinae*), which differ in genomic structure, cell tropism, and in their host–switching capacity. Indeed, cross–species transmission is repeatedly reported for members of the *Alphaherpesvirinae* and the *Gammaherpesvirinae* subfamilies [3,4,5,6]. Conversely, in betaherpesviruses, inter–species transmissions are rare events, indicating that, in natura, these viruses are restricted to their natural host [7,8,9,10] (see below).

In this review, we focus our attention on the evolution and virus–host relationships of primate–infecting cytomegaloviruses (CMVs). These viruses are classified in the genus *Cytomegalovirus* in the *Betaherpesvirinae* subfamily [1]. Up to date, 11 CMVs have been recognized as species by the International Committee on Taxonomy of Viruses (ICTV, https://talk.ictvonline.org/, 2018b release, MSL #34), although the complete genome is only available for nine of them (Table 1).

**Figure 1:**
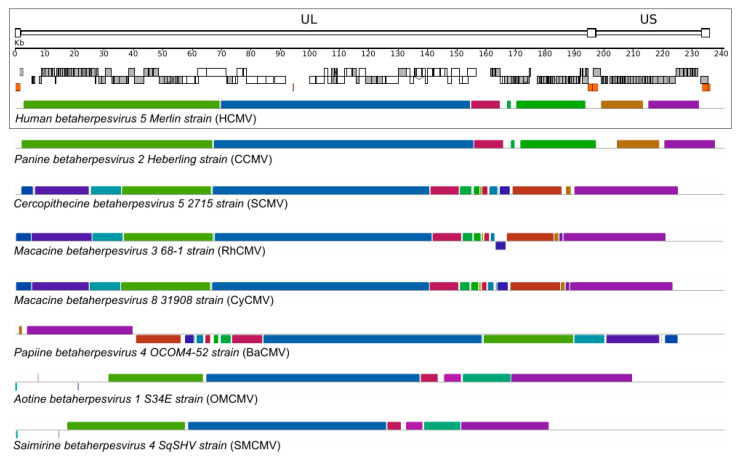
**Primate CMV genome organization**. A schematic representation of the HCMV genome is shown in the upper panel. Annotated coding regions are represented as boxes (white: core genes, gray: non–core genes); repetitive elements are shown as orange boxes. Colored similarity plots with primate CMV genomes are also shown. The whole–genome CMV alignment was obtained with progressive MAUVE [16,17]. Each genome is laid out in a horizontal track and each colored block delimits a genome region that aligns to part of another genome (presumably homologous and free from internal rearrangements) and thus represents a locally collinear block. When the similarity plot points downward it indicates an alignment to the reverse strand of the genome. *Genome IDs: HCMV*, *NC_006273; CCMV, NC_003521; SMCV, NC_012783; RhCMV, NC_006150; CyCMV, NC_033176; BaCMV, NC_027016; OMCMV, NC_016447; SMCMV, NC_016448*.

Typically, CMVs display large genomes, exceeding 200 Kb in length, with more than 160 protein–coding genes [2]. Human cytomegalovirus (HCMV, species *Human herpesvirus 5*) has a genome about 235 Kb in size, one of the largest among herpesviruses and, generally, among human–infecting viruses [11]. CMV genomes consist of unique long (UL) and unique short (US) segments, each of which is flanked by inverted repeats (RL and RS) (Figure 1). Protein–coding genes occupy the great majority of CMV genomes. As detailed below, genes located in the central portion of the UL region constitute a cluster of *core* genes that have homologs in other herpesviruses and perform core functions. The other genes, referred to as *non–core*, are primarily identified in betaherpesviruses or are species–specific. These genes are mainly involved in viral persistence and latency, cellular tropism, and host immune response modulation [2,11,12,13,14]. Moreover, CMVs encode a number of miRNAs that regulate host cellular and viral transcriptions to favor viral infection and inhibit the host’s immune response [15].

HCMV is a ubiquitous human pathogen, which infects 56–94% of the global population [18]. The seroprevalence increases with age and varies with socioeconomic status and geographical location, with developing countries having the highest prevalence [18]. In most immunocompetent individuals, HCMV infections are asymptomatic, as viral replication is controlled by the host immune system; very often, such infections are undiagnosed, as symptoms are ambiguous and relatively mild [19]. However, in analogy to other herpesviruses, HCMV establishes lifelong persistence as a latent infection. Virus latency is characterized by absent or low–level virus replication [20], with the viral genome persisting in a dormant form predominantly in the CD34+ hematopoietic progenitor cell population in the bone marrow. Virus reactivation, as well as reinfection, is common, especially for susceptible groups, such as immunocompromised patients, pregnant women, newborns, and elderly people [19,21]. In immunocompromised individuals, including transplant recipients and AIDS patients, HCMV primary infection, reinfection, or reactivation can cause a life–threatening disease that affects many organs and results in considerable morbidity and mortality. HCMV is also the most common infectious cause of birth defects. In high–income countries, where maternal seroprevalence is relatively low, rates of congenital HCMV infection are around 0.6 to 0.7% of live births. However, in resource–limiting settings, the prevalence can be as high as 1.5% [22]. Congenital HCMV infection can result in mild to severe clinical outcomes in the developing immune naive fetus. Lifelong sequelae requiring long–term rehabilitation are common in congenitally infected infants and include sensorineural hearing loss, cognitive impairment, and/or vision defects [22]. Furthermore, some lines of evidence suggest that HCMV plays a role as an oncomodulator in the development of different types of cancer [20,23,24] and that it is involved in the pathogenesis of atherosclerosis [25], as well as of inflammatory bowel disease [26]. Finally, HCMV is thought to cause or accelerate immunosenescence in the elderly population, a condition also known as immune aging [27,28].

Thus, some aspects of HCMV pathogenesis are closely linked to the ability of the virus to persist latently in the host. Lifelong persistence is achieved by HCMV through a number of mechanisms that ensure escape from the humoral and cellular host immune responses [29]. Specifically, during their evolutionary history, CMVs (and herpesviruses in general) have acquired new coding genes, which, on the basis of sequence or structure similarity, were most likely captured from their hosts via lateral transfer [30]. These include homologs of host cytokines and their G coupled receptors [30,31,32] which are frequently involved in duplications and deletions in CMV genomes so as to generate species–specific arrays of immunomodulatory proteins. These undermine the host antiviral immunity, eventually reducing immune recognition of the pathogen through molecular mimicry and other mechanisms [29].

As anticipated above, CMVs infect a wide variety of NHPs (Table 1) [7,8,9,10,11,33,34,35,36,37,38,39,40]. As detailed in the following section, NHP–infecting CMVs share with HCMV a similar genomic organization and coding content, as well as the course of viral infection (lifelong persistence). As in humans, CMVs that infect other primates are generally widespread, reaching high seroprevalence levels. For example, in breeding colonies of rhesus macaques, almost the totality of animals are seropositive for RhCMV (rhesus macaque CMV) before the first year of age. High rates of infection were also reported for other NHPs in the wild [10,33,41]. Overlaps between HCMV and NHP CMVs are also observed in terms of infection outcome and clinical presentation. Thus, adult macaques manifest symptoms only when immunocompromised, as a result of primary infection or viral reactivation. In simian AIDS, CMV can produce end–organ disease in different tissues, reflecting HCMV pathogenesis in AIDS subjects [42]. Finally, different studies indicated that congenital RhCMV transmission recapitulates congenital HCMV pathogenesis, with spontaneous abortions or fetuses with brain and neurological defects [42].

**Table 1:** List of known primate–infecting CMVs.

Primate species		CMV species	Reference
*Scientific name*	Common Name	*Official name* /Common Name (Abbreviation)	
**Great apes**			
*Homo sapiens*	Human	Human betaherpesvirus 5 *	[11]
*Pan paniscus*	Bonobo	Pan paniscus cytomegalovirus (PpanCMV1, PpanCMV2)	[33]
*Pan troglodytes*	Chimpanzee	*Panine betaherpesvirus 2* (CCMV) *	[34]
*Pan troglodytes ellioti*	Nigeria–Cameroon chimpanzee	*Panine betaherpesvirus 2* (CCMV1, CCMV2)	[33]
*Pan troglodytes schweinfurthii*	Eastern chimpanzee	*Panine betaherpesvirus 2* (CCMV1, CCMV2)	[33]
*Pan troglodytes troglodytes*	Central chimpanzee	*Panine betaherpesvirus 2* (CCMV1, CCMV2)	[33]
*Pan troglodytes verus*	West African chimpanzee	*Panine betaherpesvirus 2* (CCMV1, CCMV2)	[35]
*Pongo pygmaeus pygmaeus*	Orang–utan	Pongo pygmaeus cytomegalovirus (PpygCMV1)	[35]
*Gorilla beringei beringei*	Mountain gorilla	Gorilla beringei cytomegalovirus (GberCMV1, GberCMV2)	[33,36]
*Gorilla beringei graueri*	Eastern lowland gorilla	Gorilla graueri cytomegalovirus (GgraCMV1, GgraCMV2)	[33]
*Gorilla gorilla gorilla*	Western lowland gorilla	Gorilla gorilla cytomegalovirus (GgorCMV1, GgorCMV2)	[35]
**Old World Monkeys**			
*Colobus guereza*	Mantled guereza	Colobus guereza cytomegalovirus (CgueCMV)	[37]
*Colobus polykomos*	Black–and–white colobus	Colobus polykomos cytomegalovirus (CpolCMV)	[7]
*Pilocolobus badius*	Western red colobus	Piliocolobus badius cytomegalovirus (PbadCMV)	[9]
*Cercopithecus aethiops*	Grivet	*Cercopithecine betaherpesvirus 5* (SCMV) *	[38]
*Cercopithecus campbelli*	Campbell’s monkey	Cercopithecus campbelli cytomegalovirus (CcamCMV)	[9]
*Cercopithecus cephus*	Moustached monkey	Cercopithecus cephus cytomegalovirus (CcepCMV)	AY728178.1
*Cercopithecus diana*	Diana monkey	Cercopithecus diana cytomegalovirus (CdiaCMV)	[9]
*Cercopithecus kandti*	Golden monkey	Cercopithecus kandti cytomegalovirus (CkanCMV)	[36]
*Cercocebus agilis*	Agile mangabey	Cercocebus agilis cytomegalovirus (CagiCMV)	AY608713.1
*Cercocebus atys*	Sooty mangabey	Cercocebus atys cytomegalovirus (CatyCMV)	[9]
*Macaca fascicularis*	Crab–eating macaque	*Macacine betaherpesvirus 8* (CyCMV) *	[8]
*Macaca mulatta*	Rhesus macaque	*Macacine betaherpesvirus 3* (RhCMV) *	[39]
*Papio anubis*	Olive baboon	*Papiine betaherpesvirus 3* (BaCMV)	[40]
*Papio cynocephalus*	Yellow baboon	Papio cynocephalus cytomegalovirus (PcynCMV)	[40]
*Papio ursinus*	Chacma baboon	*Papiine betaherpesvirus 4* (BaCMV) *	[43]
*Mandrillus leucophaeus*	Drill	*Mandrilline betaherpesvirus 1* (MleuCMV) *	[43]
*Mandrillus sphinx*	Mandrill	Mandrillus sphinx cytomegalovirus (MsphCMV)	[35]
**New World Monkeys**			
*Sapajus apella*	Tufted capuchin	Sapajus apella cytomegalovirus (SapeCMV)	[10]
*Cebus albifrons*	White–fronted capuchin	*Cebine betaherpesvirus 1* (CalbCMV)	[10]
*Cebus capucinus*	White–headed capuchin	*Cebine betaherpesvirus 1* (CcapCMV)	[10]
*Saimiri boliviensis boliviensis*	Black–capped squirrel monkey	Saimiri boliviensi cytomegalovirus (SbolCMV)	[10]
*Saimiri sciureus*	Squirrel monkey	*Saimiriine betaherpesvirus 4* (SMCMV) *	[38]
*Saimiri sciureus albigena*	Colombian common squirrel monkey	Saimiri albigena cytomegalovirus (SalbCMV)	[10]
*Aotus trivirgatus*	Three–striped night monkey	*Aotine betaherpesvirus 1* (OMCMV) *	[38]
*Aotus vociferans*	Spix’s night monkey	Aotus vociferans cytomegalovirus (AvocCMV)	[10]
*Aotus nancimaae*	Nancy Ma’s night monkey	Aotus nancymaae cytomegalovirus (AnanCMV)	[10]
*Pithecia pithecia*	White–faced saki	Pithecia pithecia cytomegalovirus (PpitCMV)	[10]
*Alouatta macconelli*	Guyanan red howler	Alouatta macconelli cytomegalovirus (AmacCMV)	[10]
*Alouatta palliata*	Mantled howler	Alouatta palliata cytomegalovirus (ApalCMV)	[10]
*Alouatta seniculus*	Venezuelan red howler	Alouatta seniculus cytomegalovirus (AsenCMV)	[10]
*Ateles paniscus*	Red–facied spider monkey	Ateles paniscus cytomegalovirus (ApanCMV)	[10]

Note: ICTV–recognized viral species are in italic; bold and asterisks denote complete genome sequences.

## 2. Diversity and Host–Specificity of Primate CMVs

The first CMV identified in a NHP was isolated in 1962 from an African green monkey (*Cercopithecus aethiops*) [44]. Thereafter, natural CMV infection was reported in a multitude of NHPs [9,10,33,35,37]. Initially, NHP CMVs were detected mostly in captive animals, but in the last few years surveys were extended to animals living in the wild, so as to generate a more accurate picture of the genetic diversity and distribution of these viruses in nature [9,10,33,35]. To date, CMVs have been detected in over 40 different primates, including great apes, as well as Old World and New World primate species, although complete genome sequences are available for a minority of these viruses (see Table 1).

Each primate species is infected by one or few species–specific CMVs. Indeed, cross–species CMV transmission has never been reported in natural settings, either between closely related species sharing the same habitat, or in NHP predator–prey systems in the wild [7,9,33]. The only limited evidence of cross–species transmission in vivo relates to very peculiar experimental settings or anecdotal reports [8,45,46,47,48]. This marked species–specificity, together with the broad distribution and the almost asymptomatic condition of infected individuals [41], suggests that co–divergence and long–standing adaptation to their hosts largely shaped the evolution of CMVs. This view is also supported by the congruence between the viral and host phylogenies, a general feature for members of the *Herpesvirales* order [49,50]. These observations do not imply, however, that host–switches did not contribute to the shaping of CMV diversity and host associations. For instance, two CMVs (referred to as CMV1 and CMV2) were described in Western chimpanzees (*Pan troglodytes verus*) and Western lowland gorillas (*Gorilla gorilla gorilla*) [33,35]. Phylogenetic analyses showed that the sequences of these viruses cluster into two different clades, both including CMVs from chimpanzees and gorillas [33,35]. These data clearly suggest a more complex evolutionary scenario of CMVs in hominines, whereby two bidirectional ancestral cross–species transmission events between hosts belonging to chimpanzee and gorilla lineages most likely occurred. Recent estimates indicated that the transmission of CMV1 from gorillas to panines occurred around 2 million years ago, whereas CMV2 crossed the species barrier from panines to gorillas approximately 1.2 million years ago [33]. Conversely, the divergence of bonobo and chimpanzee CMV1 and CMV2 were synchronous with the divergence of their hosts (0.87 million years ago), indicating subsequent virus–host co–divergence [33].

Whereas CMV host–specificity is very strict in vivo, replication in different host cells in vitro seems to be slightly more permissive, with reported cases of cell infection for closely related host species only [51,52,53] but not for phylogenetically distant hosts [54]. For instance, HCMV can replicate at low levels in chimpanzee primary fibroblasts [53], but other NHP cells do not support the production of infectious virions [55,56,57,58].

The molecular mechanisms responsible for this strict host–specificity are not fully understood. As mentioned above, CMVs display very large genomes, organized into two covalently linked segments [34]. As shown in Figure 1, the overall genome architecture is conserved among primate CMVs. The majority of *core* genes, which encode essential proteins involved in DNA replication, virion assembly and maturation, are confined to the central shared collinear block (Figure 1). Clearly, *core* genes are highly conserved in sequence among CMVs. For example, HCMV sequence identity ranges from 60 to 90% with chimpanzee CMV (CCMV), and from 50 to 82% with RhCMV [41]. Conversely, *non–core* genes are less conserved in sequence and number, with variability observed between closely–related CMV species and, sometimes, even among isolates of the same species (see below). These genes are often dispensable for viral growth in cell culture [13,14], but play important roles during infection in vivo [13,41,59]. In particular, a significant proportion of *non–core* genes have evolved to elude and subvert the host immune response [41,59]. It is worth mentioning here that adaptation to cell culture is a common feature of CMVs. This is due to the fact that viral mutants are selected during isolation and passage in fibroblasts or other cell types, where no pressure is exerted by the host adaptive immune response [12]. Thus, mutations (including relatively large deletions and rearrangements) occur in genomic regions that are not essential for viral replication and the process originates lab–adapted strains, which differ in gene content from clinical isolates [12].

Both commonalities and differences among primate CMV genomes most likely represent key elements to understand the adaptation of these viruses to different, but closely related, primate hosts. One of the best examples of host specificity of primate CMVs is the relationship between RhCMV, CyCMV (cynomolgus macaque cytomegalovirus), and their respective hosts. The two viruses are closely related, they possess collinear genomes and show a high level of genetic identity, mirroring the close phylogenetic relationship among their hosts (i.e., Indian–origin rhesus macaques and Mauritian–origin cynomolgus macaques) (Figure 1) [8]. Despite these similarities, in vivo cross–infection has not been documented yet, although several lines of evidence suggest that CyCMV can infect different hosts and different cell types in vitro [52,53,60,61]. At the same time, different lab–adapted CyCMV and RhCMV strains have shown the ability to infect different host cells [8,51,61]. This peculiar characteristic of lab–adapted strains has allowed the identification of specific genes that may have a role in cross–species infection [8,60]. In particular, an engineered strain of RhCMV, with restored expression of *UL36* or *UL128*, together with *UL130*, can infect cynomolgus macaques both in vitro and in vivo [8]. The specific function of these genes underlies their role in cross–species infections. pUL36 plays a role in the inhibition of apoptosis by binding to caspase–8 and preventing its activation [62] pUL128 and pUL130 interact with each other and form a complex with other viral envelope glycoproteins to facilitate entry in non–fibroblast cell types [63]. Overall, these results indicate that different mechanisms contribute to species–specificity and that cell culture passages can have an impact in studying cross–species transmission.

Other interesting candidates as genetic determinants of host–specificity in vivo are the members of gene families encoding viral CXC chemokine ligand–like proteins (vCXCL) and genes encoding viral G protein–coupled receptor–like proteins (vGPR). These genes are found to be tandemly repeated in primate CMVs, and each viral genome encodes a different number of them [64]. Although their function is not fully understood, it has been proposed that their diversity in terms of number and sequence is associated with viral infection in vivo, likely by a different modulation of the host immune response [64,65].

However, as mentioned above, some CMVs such as HCMV display species–specificity even in cell culture i.e., in the absence of the host adaptive immune response. Such specificity is mainly driven by post–entry events [56,57,58,66,67], indicating that, in order to become one of the most successful human pathogens, HCMV must have adapted to efficiently complete its infectious cycle in human cells. As expected, the closest relative of HCMV is CCMV, which displays a strong conservation in terms of genome organization and sequence (Figure 1) [34]. Thus, comparison of the genomes of these two viruses can provide relevant information on the events underlying HCMV adaptation to our species. Adaptation to a new host is often accompanied by episodes of positive selection, a situation whereby amino acid replacements accumulate at a faster rate than expected based on the rate observed at synonymous sites [68]. A recent genome–wide scan of positive selection identified changes that most likely arose during HCMV adaptation to the human host. Several signals of positive selection were found to be located within *core* genes [69]. In particular, evidence of selection was found in genes encoding capsid components and tegument proteins that drive important steps in virion maturation and assembly. Overall, these results suggest polygenic adaptation of HCMV to its human host. However, analysis of selected sites in a core viral enzyme, the primase (pUL70), indicated that two amino acid changes, which most likely arose during the emergence of HCMV as a species, decrease rather than increase replication in human cells. In human fibroblasts, mutant viruses that recapitulate the amino acid state observed in non–human infecting CMVs had higher fitness than the wild–type HCMV strain [69]. Although this finding may seem counter–intuitive, the evolution of mechanisms that reduce or control viral replication in the host might be necessary to decrease virulence and ultimately promote long–standing host–pathogen associations. A similar hypothesis had previously been put forward by Dunn and co–workers, when a functional assessment of HCMV coding potential indicated that the deletion of some genes (e.g., *UL10*, *UL23*, and *US16*) increased replication in specific cell types. The authors thus suggested that a temperance effect is necessary to promote the long–term co–existence of the virus with its host [70].

## 3. High Population–Level Diversity of Circulating HCMV Strains

The first complete HCMV genome sequence, that of the high–passage strain AD169, was obtained in 1989, from a plasmid library [71]. More than a decade later, other laboratory strains and clinical isolates were grown in cell culture and sequenced [11,12,70]. These and other early efforts started to reveal that the genetic diversity of HCMV is high and that mutations frequently arise in cell culture [72]. More recently, the development of high–throughput technologies has allowed the sequencing of a sizable number of HCMV strains, either directly from clinical specimens or after a small number of passages [73,74,75,76,77,78]. To date, more than 300 complete HCMV genomes are available in public databases. However, the geographic origin of these sequences is highly biased, with the majority of samples coming from Europe and USA, a few from Israel, and one from Asia. As for Sub–Saharan Africa, where the virus is highly prevalent [18], the first four complete HCMV genome sequences were obtained in 2019 [74].

**Figure 2:**
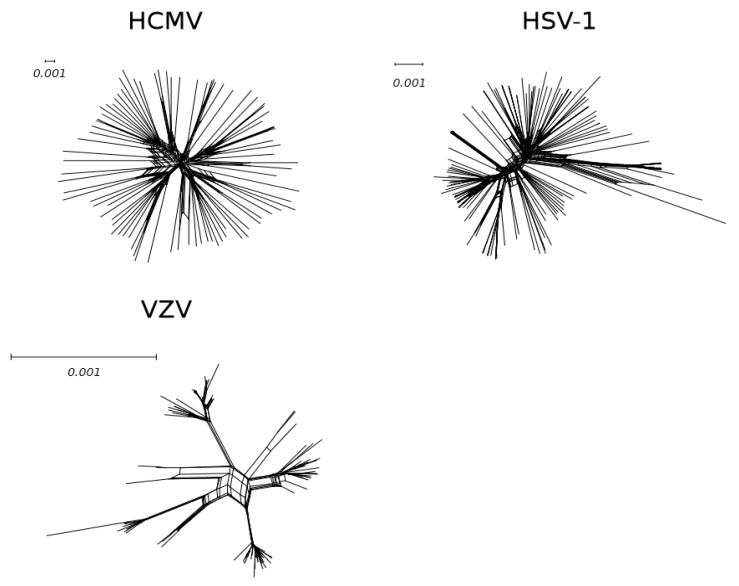
**Genetic diversity of human herpesviruses.** Neighbor–net split networks of 84 unpassaged HCMV (Supplementary Table S1), 140 HSV–1 [79], and 110 VZV [80] genomes. A phylogenetic network is a graph to visualize the evolutionary relationships (edges) among taxa (nodes). In particular, a split network represents incompatibilities within a data set by combining the results of different phylogenetic trees. Neighbor–net split networks of all whole genome sequences were constructed with SplitsTree v4.13.1 [81] using uncorrected p–distances and all polymorphic sites, after removing gap sites.

Parallel advances in the sequencing of other human herpesviruses allowed comparison among these common human pathogens. It was thus shown that, at the population level, HCMV is significantly more genetically diverse than HSV–1 (Herpes simplex virus), EBV (Epstein–Barr virus), and VZV (Varicella–zoster virus) [75]. If the scale is considered, the difference between the HCMV and HSV–1 trees is impressive, and even more so if HCMV is compared to VZV (Figure 2).

Another notable difference among human herpesviruses is the degree of geographic structuring of viral populations. At one extreme stand VZV and EBV, which display a clear spatial distribution of genetic diversity [82,83,84,85]. For instance, VZV clades 1, 3, and 6 are mainly transmitted in Europe, North America, and Australia, whereas clade 2 is primarily Asian [83]. Other human herpesviruses such as HSV–1 and HSV–2 display limited geographic stratification of genetic diversity, and this is also the case for HCMV [74,75,79,86,87]. On one hand, the absence of geographic clustering might be partially due to the skewed origin of available HCMV sequences. For instance, in the case of HSV–2, a viral lineage that is mainly transmitted in Africa was discovered only recently [88], suggesting that, likewise, a portion of HCMV diversity remains unknown, either in Africa or in other continents. On the other hand, different factors related to distinct aspects of viral biology most likely account for the geographic structuring of genetic diversity or lack thereof. In the case of VZV, recent data indicated that viral lineage extinction may be common for this virus, most likely as a consequence of its epidemiology, which is mainly characterized by large chickenpox outbreaks among children (before the introduction of a vaccine) [80]. This feature may contribute to the local emergence of viral lineages and favor geographic segregation. Human herpesviruses other than VZV are not characterized by epidemic spread, though. Recent analyses, however, indicated that HCMV and HSV–1 have a different recombination pattern compared to EBV. In particular, the two former viruses are essentially freely recombining, with only limited regions of linkage disequilibrium (LD) throughout their genomes [85,87]. Conversely, despite abundant recombination, EBV shows extensive genome–wide LD [85]. Because sites in LD are enriched in EBV genes that encode immunogenic proteins, Wegner and coworkers suggested that viral population structure is determined by local adaptation to human populations [85]. In summary, these data suggest that, although a wider sampling of HCMV diversity may in the future reveal some level of geographic clustering, most HCMV stains derive from a highly recombining panmictic population distributed worldwide.

Indeed, recombination stands out as a major driver of HCMV genetic diversity [73,75,87,89]. Recombination clearly requires that the same individual is infected by more than one strain and, as mentioned above, mixed HCMV infections are very common. Based on these observations, as well on their data showing that a minority of genes involved in immune evasion or cell entry are highly divergent and non–recombining, Lassalle and coworkers [87] suggested that HCMV has evolved strategies to promote super–infection and, consequently, recombination. This would serve the purpose of reassorting such highly diverse regions and to ensure efficient immunoevasion.

Clearly, recombination is not the only force shaping HCMV diversity, and genetic polymorphism is not evenly distributed across the genome [75]. Genes encoding viral glycoproteins have long been recognized among the most variable in HCMV strains [90,91]. More recent studies that systematically analyzed sequence diversity along HCMV genomes indicated that glycoproteins and proteins involved in immunomodulation tend to be less constrained and to display the strongest signals of positive selection [69,75,76,86,92]. Also, selection is stronger in protein regions exposed on the virion surface and, for viral proteins expressed at the host cell membrane, in the extracellular domains [69]. Overall, these observations suggest that the selective pressure exerted by the host immune system has likely played a major role in the shaping of genetic diversity among circulating HCMV strains. In line with this view, several sites targeted by positive selection are located within epitopes recognized by human antibodies or in protein regions that directly interact with host molecules involved in immune response [69,75]. These features are consistent with an ongoing genetic conflict between HCMV and the human immune system.

However, recent analyses also detected hotspots of diversity and positive selection that are more difficult to reconcile with an underlying selective pressure. One of these is the furin cleavage site of glycoprotein B (gB) [69,93]. Positively selected sites in this region affect the kinetic of cleavage by furin and were suggested to modulate viral spread in different tissues, although evidence for this is presently lacking [69,93]. Another unusual selection signature was reported within the signal peptides of several HCMV proteins [69]. Functional characterization of one of these indicated that the sequence of the signal peptide of the pUL144 glycoprotein determines the timing of cleavage by the signal peptidase and the dynamics of pUL144 intracellular trafficking. The phenotypic effect of such changes in sorting dynamics is presently unclear but, by analogy with the HIV–1 envelope protein [94], we recently suggested that variation within the signal peptide might affect protein folding or glycosylation, ultimately modulating antigenic properties [69]. If this were the case, HCMV signal peptide diversity might represent an additional layer of control over host immune surveillance.

## 4. Frequency and Origin of Gene–Disrupting Mutations in HCMV Strains

Another unexpected feature that emerged from the analysis of several HCMV genomes is the frequency with which disruptive mutations occur in different genes. These mutations, which include single nucleotide substitutions, small frameshifting insertions/deletions, and large genomic deletions, are predicted to have deleterious effects on gene expression and protein function. This phenomenon is well described in laboratory passaged HCMV strains [95,96], but several lines of evidence suggest that it also occurs in clinical isolates [75,77,96,97].

A peculiar feature of these mutations is that specific genes and gene families are usually affected. These include the *UL11*, *RL1*, *US6*, and *US12* families [75] and most mutated genes are dispensable for viral growth in vitro [75], but possibly have a role in cell tropism [96]. Disrupting mutations have been found in other genomic regions, but they seem to be sporadic [96].

The emergence of deleterious mutations in laboratory strains can be explained by viral adaptation to several cell–cultured passages [95], but for clinical samples the explanation is likely to be more complex. In fact, some of these disruptive changes (both indels or point mutations) are shared among different strains, suggesting that they were already present in their common ancestor [96].

**Figure 3:**
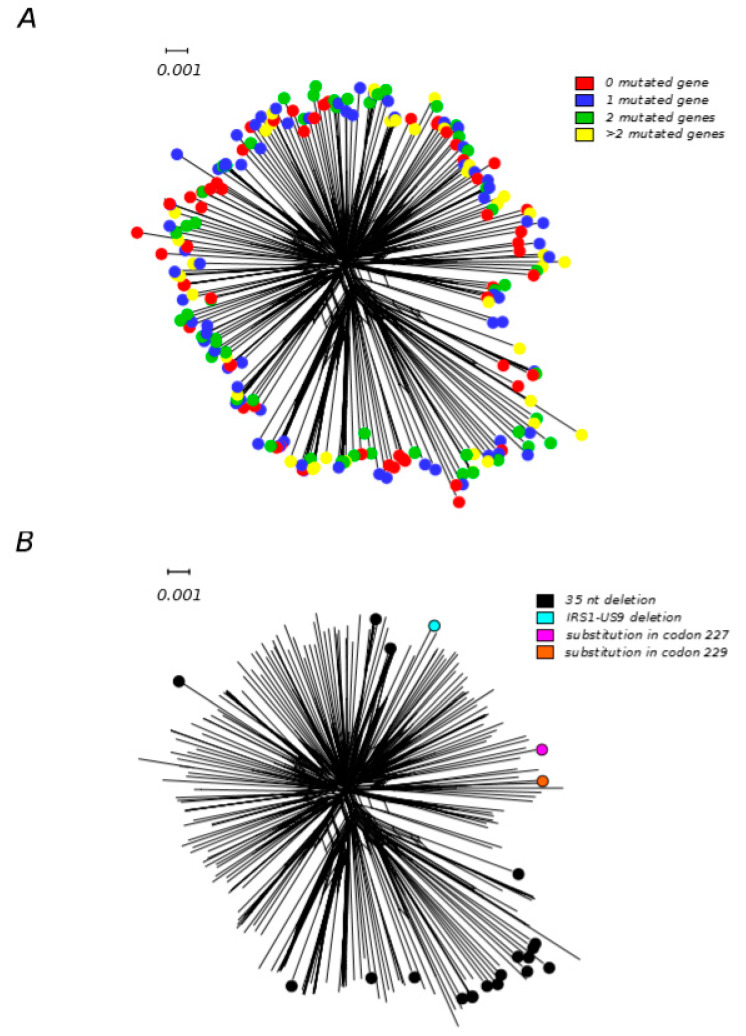
Gene disrupting mutations in HCMV strains. Neighbor–net split network of 233 HCMV genome sequences. (**A**) Strains are color–coded on the basis of the number of mutated genes. (**B**) Strains are color–coded depending on the type of US9 mutation (Supplementary Table S1). Neighbor–net split networks of genomic sequences were constructed with SplitsTree v4.13.1 [81] using uncorrected p–distances and all polymorphic sites, after removing gap sites.

To further explore this scenario, we retrieved gene annotation for 233 HCMV genomes from the GenBank database (Supplementary Table S1) and we analyzed the disrupted gene frequency and the number of passages in cell culture. Among these strains, 84 were annotated as unpassaged, the remaining went through one to several passages. One–hundred–and–seventy out of 233 strains (58 unpassaged, 112 with at least one passage) had at least one disrupted gene, reinforcing the idea that this phenomenon is not dependent on laboratory adaptation, as previously suggested [73,75]. For many genes, different genomic variations (i.e., insertions, deletions, and single nucleotide mutations) were identified, indicating that disruptive mutations arose multiple times and are not only the result of common ancestry. We then analyzed the number of genes and which genes were mutated. Ninety–seven genomes carried more than one truncated coding sequence (Figure 3A), resulting in several different combinations of gene losses in HCMV strains. As previously shown [75], the most frequently mutated genes were *UL9*, *RL5A*, *UL1*, *RL6*, and *US9*. So we tested whether strains with the same mutated gene (regardless of the specific mutation they carry) shared a common ancestor and, consequently, a common evolutionary history. To that purpose, we applied two different methods that, by taking phylogenetic uncertainty into account, evaluate whether different viruses from a population follow or not independent evolutionary histories [98]. In particular, we applied one method based on reconstructing phylogenetic trees (Slatkin–Maddison test) and one genetic–distance–based method (Nearest–neighbor statistic) [98,99] on all 233 genome sequences. These sequences were divided between the ones carrying all different mutations in one of the mutated genes and all the other strains. This analysis was repeated for the five most frequent mutated genes. We found that genomes carrying four different *US9* detrimental mutations shared a more similar evolutionary history compared to what would be expected by chance (*p* < 0.01 for both tests) (Figure 3), suggesting that the genetic background influences the ability to tolerate a non–functional *US9* gene. The best characterized function of the pUS9 protein is to target MAVS and the STING–TBK1 signaling pathway to inhibit IFN–β expression and, consequently, the downstream antiviral responses [100]. This function is, however, partially redundant with those exerted by other viral proteins (i.e., pUL83, pUL82, and pUL122) [101]. pUS9 might also play antiapoptotic functions, by inhibiting the short form of MAVS. Antiapoptotic activity is also exerted by pUL37 (also known as vMIA), which binds to and recruits Bax to mitochondria [102]. It is thus possible that, depending on their genetic make–up, some HCMV strains are particularly efficient at inhibiting apoptosis and IFN–β expression, and that the action of pUS9 is therefore dispensable. However, the maintenance in the HCMV population of multiple genes with partially overlapping functions, together with sustained recombination rates, is likely to confer mutational robustness and important adaptive advantages.

## 5. Conclusions and Future Perspectives

For a long time, the large size of CMV genomes has posed challenges to their sequencing. The advent of high–throughput technologies has thus particularly benefited the field of CMV genomics. Such technologies not only allow the rapid sequencing of long genomic regions, but also offer the possibility to obtain complete genome sequences from little amounts of viral DNA. Thus, the direct sequencing from patients’ samples has bypassed the problems associated with the in vitro passaging of HCMV strains, so that hundreds of genomes of clinical isolates were obtained [73,74,75,77,78,97]. In parallel, field work has extended knowledge about CMVs hosted by NHPs living in the wild [9,10,33,35]. These advances have provided invaluable insight into the evolutionary history of primate–infecting CMVs. The emerging picture indicates that, with the exclusion of *core* genes, CMV genomes are relatively plastic and diverse in terms of gene content, both at the inter– and at the intra–species level. Such variability is most likely a key element underlying the strict species–specificity of these viruses, as well as their ability to persist lifelong and with relatively little damage to their hosts. Nonetheless, *core* genes, despite their strong conservation, also represent a target of adaptive evolution and subtle changes in their coding sequence contribute to the success of CMV infection.

However, large gaps of knowledge remain, first and foremost the role of HCMV genetic diversity in the development of human disease. Several studies have indicated that mixed HCMV infection is associated with poor clinical outcome in immunocompromised individuals [103,104,105]. Nonetheless, the role of specific viral variants in the progression and severity of disease in these individuals, as well as in congenitally infected infants, remains unknown. Congenital HCMV infection results in a wide variety of clinical outcomes [22], suggesting a complex interplay between viral and host factors. The increasing ability to sequence viral genomes from infected newborns, as well as the collection of related clinical data might in the future allow the identification of viral markers (if any) associated with short–term and long–term outcomes. Likewise, deeper knowledge of worldwide HCMV diversity will be instrumental to the development of a much needed effective vaccine.

## Supplementary Materials

The following are available online at https://www.mdpi.com/2076-2607/8/5/624/s1, Table S1: List of HCMV strains analyzed in this study.
