# Supplementary material for: Evolution and Genetic Diversity of Primate Cytomegaloviruses
Source: Microorganisms. 2020 Apr 25;8(5):624. doi: 10.3390/microorganisms8050624 (PMC7285053; doi:10.3390/microorganisms8050624)
Supplement: Supplementary file 1 [file microorganisms-08-00624-s001.pdf]

Supplementary Table 1: List of HCMV strains analyzed in this study.

Strain name	GenBank ID	Mutated genes
3301*	GQ466044	
AF1*	GU179291	*RL6, UL9*
BE/21/2010*	KC519322	*RL5A, UL9, UL150*
HANChild1*	KY490071	*RL5A*
HANChild2&3*	KY490072	*RL5A, US9*
HANChild4*	KY123649	
HANRTR10*	KY490078	*UL1, US7*
HANRTR1A*	KY490073	*UL9, US6*
HANRTR1B*	KY490074	
HANRTR2*	KY123650	
HANRTR4*	KY123651	*UL1*
HANRTR5*	KY123652	
HANRTR6*	KY490075	
HANRTR8*	KY490076	*RL6, UL9, UL30, US8*
HANRTR9*	KY490077	*UL1, UL9*
HANSCTR10*	KY490084	*RL1, RL6, UL142*
HANSCTR11A*	KY490085	*UL9*
HANSCTR11B*	KY490086	*RL5A, RL6*
HANSCTR12*	KY490087	*UL1*
HANSCTR13*	KY490088	*UL1*
HANSCTR1A*	KY490079	*UL43, US7*
HANSCTR1B*	KY490080	*RL5A*
HANSCTR2*	KY490081	*RL5A, UL9*
HANSCTR4*	KY123653	*UL1, UL9*
HANSCTR8*	KY490082	*UL1*
HANSCTR9*	KY490083	
JER1070*	KR534199	
JER2002*	KR534201	*RL5A, UL9*
JER2282*	KR534202	*RL6*
JER3230*	KR534203	*UL9*
JER3855*	KR534204	*RL5A*
JER4035*	KR534205	
JER4041*	KR534206	*UL111A*
JER4053*	KR534207	
JER4559*	KR534208	*RL5A, UL9*
JER4755*	KR534209	*RL5A, UL9, UL40*
JER5268*	KR534210	*RL5A, UL1, US7*
JER5409*	KR534211	
JER5550*	KR534212	*RL6, UL9*
JER5695*	KR534213	*UL9, UL111A*
JHC*	HQ380895	*UL140, UL1, UL131A*
JP*	GQ221975	*RL5A, UL111A*
LUS193*	MK290742	
LUS243*	MK422176	
LUS248*	MK290743	
LUS283*	MK290744	

NAN1LA*	KU550087	*RL6, US9*
NAN2LA*	KU550088	
NAN4LA*	KU550089	*RL6, UL9, US9*
NANU*	KU550090	*RL5A*
PAV1*	KJ361959	*RL5A*
PAV11*	KJ361965	
PAV12*	KJ361966	*RL5A*
PAV16*	KJ872539	*RL5A, UL9, UL111A*
PAV18*	KJ872540	
PAV20*	KJ872541	*RL5A*
PAV21*	KJ872542	*UL1, UL9, RNA4.9*
PAV23*	KJ361967	*RL1, RL5A*
PAV24*	KJ361968	*RL5A*
PAV25*	KJ361969	*UL9, UL150*
PAV26*	KJ361970	*UL1, UL9*
PAV31*	KY490061	*RL5A, UL13*
PAV32*	KY490062	*RL6*
PAV4*	KJ361960	*RL5A, UL150, US7*
PAV5*	KJ361961	*US8*
PAV6*	KJ361962	*RL1, US7*
PAV7*	KJ361963	*US7*
PAV8*	KJ361964	*UL9*
PRA1*	KY490063	*UL1, UL9*
PRA2*	KY490064	*UL1, UL9, UL15A*
PRA3*	KY490065	*UL1*
PRA4*	KY490066	
PRA5*	KY490067	*UL1, UL9, UL30, UL111A*
PRA6*	KY490068	*RL5A, UL1, UL9*
PRA7*	KY490069	*RL5A, UL111A*
PRA8*	KY490070	
U11*	GU179290	*UL9*
U8*	GU179288	
UK/Lon1/Blood/2013*	KT726947	
UK/Lon6/Urine/2011*	KT726949	
UK/Lon7/Urine/2011*	KT726950	
UK/Lon8/Urine/2012*	KT726951	
UKNEQAS2*	KT634296	*RL6, RL9*
VR1814*	GU179289	
3157	GQ221974	*RL13, UL40, UL128*
6397	JX512197	*RL13, UL9, UL130, UL131A, IRS1, US1, US2, US3, US6, US7, US8, US9*
2CEN15	KJ361948	*UL97*
2CEN2	KJ361946	*UL1, UL9*
2CEN30	KJ361949	*UL1, UL9, UL14*
2CEN5	KJ361947	*RL5A*
AD169	FJ527563	*UL144, UL142, UL141, UL140, RL5A, RL13, UL131A, IRS1, US1, US2*
BE/1/2010	KP745677	*RL5A, US9*
BE/1/2011	KP745650	*UL1, UL9*
BE/1/2012	KP745699	*UL1, UL136*

BE/10/2010	KC519320	*UL9*
BE/10/2011	KP745639	*UL1*
BE/10/2012	KP745649	
BE/11/2010	KC519321	*RL5A, UL1*
BE/11/2011	KP745684	*RL6*
BE/11/2012	KP745680	*UL150*
BE/12/2010	KP745694	*RL5A*
BE/12/2011	KP745683	*R, US9*
BE/12/2012	KP745688	*RL6*
BE/13/2010	KP745645	*US9*
BE/13/2011	KP745657	*RL5A, UL1, UL9, US7*
BE/13/2012	KP745707	*UL9*
BE/14/2010	KP745721	*RL6, UL9, UL40*
BE/14/2011	KP745671	*UL9, UL11, US6*
BE/14/2012	KP745658	*RL6, UL9, US7*
BE/15/2010	KP745638	*RL1, UL9, UL111A*
BE/15/2011	KP745720	*RL5A, UL9*
BE/15/2012	KP745693	*UL150, US7*
BE/16/2010	KP745716	*UL1, UL111A, US9*
BE/16/2012	KP745665	*UL150*
BE/17/2010	KP745727	*RL5A, RL12, UL111A*
BE/17/2011	KP745689	*UL1, UL9*
BE/18/2010	KP745647	
BE/18/2011	KP745668	
BE/19/2010	KP745712	*UL9, UL136*
BE/19/2011	KP745654	*RL6, UL9*
BE/2/2010	KP745717	*UL9, UL111A*
BE/2/2011	KP745652	
BE/2/2012	KP745710	*RL5A, UL133*
BE/2/2013	KP745656	*RL5A, UL128, US9*
BE/20/2010	KP745662	*UL9*
BE/20/2011	KP745698	*US7*
BE/21/2011	KP745702	
BE/22/2010	KP745640	
BE/22/2011	KP745653	*RL6, UL9*
BE/23/2010	KP745697	*UL1, UL9*
BE/23/2011	KP745675	*UL9*
BE/24/2010	KP745679	*UL9*
BE/24/2011	KP745711	
BE/25/2010	KP745678	*UL111A*
BE/26/2010	KP745719	*UL150*
BE/26/2011	KP745703	
BE/27/2010	KC519323	*RL5A, UL1, UL9, UL111A*
BE/27/2011	KP745696	*UL9*
BE/28/2010	KP745676	*RL5A, UL9*
BE/28/2011	KP745669	*UL40*
BE/29/2010	KP745714	
BE/29/2011	KP745672	*RL5A, UL9*
BE/3/2010	KP745655	*RL5A, UL9*
BE/3/2011	KP745659	

BE/3/2012	KP745692	*US13*
BE/30/2010	KP745726	*US9, US27*
BE/30/2011	KP745670	*UL150*
BE/31/2010	KP745644	
BE/31/2011	KP745641	
BE/32/2010	KP745634	*RL5A, US9*
BE/32/2011	KP745704	*UL150*
BE/33/2010	KP745661	*RL6, UL9, US9*
BE/33/2011	KP745674	
BE/34/2011	KP745690	
BE/35/2011	KP745713	*UL142, US9*
BE/36/2011	KP745687	*RL6, UL9, UL40*
BE/37/2011	KP745723	*RL6*
BE/38/2011	KP745705	*RL6, UL9*
BE/39/2011	KP745686	*UL9, UL111A, US9*
BE/4/2010	KP745728	*RL5A, UL111A*
BE/4/2011	KP745700	
BE/4/2012	KP745724	*RL5A*
BE/40/2011	KP745722	*RL6*
BE/41/2011	KP745706	*UL9, UL111A*
BE/42/2011	KP745673	*RL5A*
BE/43/2011	KP745681	*RL5A, RL6, UL133*
BE/44/2011	KP745715	*RL5A, UL150A, IRS1, US9*
BE/45/2011	KP745633	
BE/46/2011	KP745682	*US9*
BE/48/2011	KP745709	*UL40 US9*
BE/49/2011	KP745725	*RL5A*
BE/5/2010	KP745663	*RL6, US9*
BE/5/2011	KP745667	
BE/5/2012	KP745635	
BE/6/2010	KP745701	*RL5A, UL9, US7*
BE/6/2011	KP745660	*UL9*
BE/6/2012	KP745695	*RL6, US9, US27*
BE/7/2011	KP745636	*RL12, UL148*
BE/7/2012	KP745666	*UL150*
BE/8/2010	KP745708	*UL9*
BE/8/2011	KP745648	*US9*
BE/8/2012	KP745646	*RL13*
BE/9/2010	KC519319	
BE/9/2011	KP745637	*UL9*
BE/9/2012	KP745651	*UL1, UL9*
CZ/1/2011	KP745718	*UL9*
CZ/1/2012	KP745642	*UL9*
CZ/1/2013	KP745691	*UL111A*
CZ/2/2012	KP745643	*US13*
CZ/2/2013	KP745664	*RL6, UL9, UL40*
CZ/3/2012	KP745685	*UL145*
Davis	JX512198	*RL5A, RL12, RL13, UL1, UL2, UL4, UL5, UL6, UL99, UL130*
DB	KT959235	*RL13, UL9*
HAN1	JX512199	

HAN11	KJ361950	*RL5A, UL150, US9*
HAN12	JX512203	*UL9*
HAN13	GQ221973	*RL5A*
HAN16	JX512204	*US7, US12*
HAN19	JX512205	*RL5A*
HAN2	JX512200	*UL1, UL6, UL7, UL8, UL9, UL30, US7*
HAN20	GQ396663	
HAN21	KJ361951	
HAN22	JX512206	
HAN27	KJ361952	*RL12, UL133*
HAN28	JX512207	
HAN3	JX512201	
HAN30	KJ361953	
HAN31	JX512208	*UL1*
HAN32	KJ361954	*UL9*
HAN33	KJ361955	*UL9*
HAN36	KJ361956	*UL20*
HAN38	GQ396662	*RL6, US9*
HAN39	KJ361957	*UL1*
HAN40	KJ361958	*RL1, US13*
HAN8	JX512202	*UL111A*
JER1289	KR534200	
JER847	KR534196	*RL6*
JER851	KR534197	*UL1, UL9, UL111A*
JER893	KR534198	*UL1, UL150*
Merlin	NC_006273	*UL128*
NL/Rot6/Nasal/2012	KT726945	
NR	KX544831	
SUB_22	KX544834	
SUB_24	KX544832	
TB40/E	KF297339	*RL13, UL128, IRS1, US1, US2*
TB40-E_UNC	KX544839	
Towne	FJ616285	*RL13, UL1, UL40, UL130, US1*
TR	KF021605	
UKNEQAS1	KJ361971	*RL5A*
VR3908	KX544833	
VR5022	KX544835	
VR5235	KX544837	
VR7863	KX544838	

Note: asterisk denotes unpassaged strains.
